# Characterization of Recombinant GMPR from *Pocillopora damicornis* and Potential Mechanisms of Cold-Induced Metabolic Adaptation

**DOI:** 10.3390/biology15110837

**Published:** 2026-05-27

**Authors:** Latha Kannan, Jaden Jones, Meghana Hosahalli Shivananda Murthy, Giovanna Ghirlanda, Judith Klein-Seetharaman

**Affiliations:** 1 School of Molecular Sciences, Arizona State University, Tempe, AZ 85281, USA; latha.kannan@asu.edu (L.K.); jkjone18@asu.edu (J.J.); mshivana@asu.edu (M.H.S.M.);; 2 College of Health Solutions, Arizona State University, Phoenix, AZ 85004, USA

**Keywords:** protein expression and purification, enzyme activity, upwelling, resilience, adaptation, climate change

## Abstract

One potential strategy to mitigate the detrimental effects of heat stress on corals is upwelling, which brings deep, cold, nutrient-rich water to the reef surface, creating transient cooling. However, cold temperatures can also stress corals, and it is, therefore, important to understand the mechanisms of both cold and heat stress responses in corals. Similar to how mammals activate thermogenic and adaptive metabolic pathways, corals may also regulate energy and redox metabolism under fluctuating environmental conditions. Guanosine monophosphate reductase (GMPR), a conserved enzyme in purine metabolism, plays a critical role in maintaining intracellular adenine and guanine nucleotide balance. To study this enzyme in corals, we expressed *Pocillopora damicornis* (PD) GMPR heterologously in *Escherichia coli* and purified the recombinant protein using nickel–NTA affinity chromatography. SDS-PAGE analysis showed a single band corresponding to the expected molecular weight, indicating high purity. Sequence alignment revealed ~70% identity with mammalian GMPR2 orthologs, suggesting evolutionary conservation of function. Structural modeling and phylogenetic analysis positioned PD GMPR between the GMPR1 and GMPR2 clades, suggesting it may represent an ancestral or functionally intermediate variant. Kinetic analysis determined Km values of 33.76 ± 6.44 μM for GMP and 17.71 ± 0.99 μM for NADPH under fixed substrate concentrations. This study provides the first biochemical characterization of GMPR, which may open the door to uncovering mechanisms of cold tolerance in corals and inform strategies to enhance coral resilience in the face of climate change.

## Simple Summary

Coral reefs are vital ecosystems that support marine biodiversity and protect coastal environments, yet they are increasingly threatened by climate change and temperature-driven bleaching. Upwelling, a process that brings deep, cold, nutrient-rich water to the ocean surface, can temporarily cool coral reefs and reduce heat stress, but it can also expose corals to sudden drops in temperature that may disrupt their metabolism. In this study, we characterize an enzyme called guanosine monophosphate reductase (GMPR) from the reef-building coral *Pocillopora damicornis*. The GMPR protein may contribute to biochemical mechanisms that help corals regulate their energy balance during cold exposure. Our findings reveal evolutionary conservation of cold response genes in corals and suggest that the metabolic adaptations GMPR catalyzes could play a role in maintaining stability under environmental stress. By expanding our understanding of the molecular pathways that support coral resilience, this research contributes valuable knowledge toward mitigating coral bleaching and protecting reef ecosystems in a changing climate.

## 1. Introduction

Coral reefs are among the most diverse and productive marine ecosystems, supporting approximately 25% of all marine species and providing essential services such as coastal protection, fisheries support, and biodiversity preservation [1]. These ecosystems are primarily formed by scleractinian corals, which build calcium carbonate skeletons in association with photosynthetic dinoflagellates of the family Symbiodiniaceae [2]. However, coral reefs are increasingly threatened by climate change, ocean acidification, and anthropogenic disturbances, leading to widespread bleaching and degradation [3]. *Pocillopora damicornis* (PD) is a reef-building coral species characterized by high morphological plasticity and relative resilience to environmental stressors [4,5,6]. It plays a key role in reef accretion and habitat formation [7] and has been widely used as a model for studying coral responses to climate change [8].

Recent mitigation strategies, including artificial upwelling and seasonal cooling, have demonstrated that periodic exposure to cooler water can enhance coral resilience to thermal extremes [9,10,11]. Upwelling, both natural and artificial, introduces deep, cold, nutrient-rich water into surface reef environments, producing transient cooling that can profoundly influence coral physiology. While elevated sea surface temperatures are the main trigger of bleaching, cold stress also poses substantial risks, especially in subtropical and temperate regions. As ectotherms, corals exhibit reduced photosynthetic efficiency, altered symbiosis, and increased bleaching under low-temperature exposure [12,13,14,15,16]. Cold-induced bleaching events, such as those in Montipora and Porites species during Hawaiian cold snaps below 18 °C, can also be destructive [17,18]. On a mechanistic level, cold stress perturbs enzyme activity and membrane fluidity, compromising photosystem II integrity, reducing energy production and affecting calcification in coral holobionts [19,20]. Beyond mitigating thermal stress, these periodic cooling events alter nutrient flux and metabolic demand, providing a natural experimental framework to study coral responses to environmental fluctuation. The metabolic adjustments observed under such conditions may parallel cold-induced adaptations in mammals, where exposure to lower temperatures activates thermogenic and redox-regulatory pathways to preserve cellular homeostasis.

The most iconic response to cold stress in humans and other mammals is the generation of heat (thermogenesis) through the action of uncoupling protein 1 (UCP1), found in brown adipose tissue (BAT), which uncouples oxidative phosphorylation and produces heat instead of ATP [21]. Two related proteins, UCP2 and UCP3, further support UCP1 action by modulating metabolism [22]. Six UCP isoforms (UCP1–UCP6) have been identified across vertebrates, each with distinct tissue distributions and metabolic functions [23,24]. UCPs belong to the mitochondrial solute carrier family SLC25 (a subfamily of the very large solute carrier family, SLC). The SLC25 family comprises 53 nuclear-encoded transporters in humans, including carriers for adenine nucleotide, aspartate–glutamate, phosphate, citrate, and redox-related shuttles [25]. Because these transport steps often become rate-limiting during metabolic stress, changes in SLC25 carrier expression or activity can have disproportionate effects on cellular homeostasis [26,27]. In mammals, cold responses involve temperature-sensitive transient receptor potential (TRP) ion channels (e.g., TRPM8, TRPA1, and TRPC5), β-adrenergic signaling, lipolysis, and mitochondrial uncoupling [28,29,30]. UCP1 is activated by free fatty acids released during cold-induced lipolysis via the cAMP–PKA pathway [21,31] and inhibited by purine and pyrimidine nucleotides (e.g., GTP, GDP, ATP, ADP, and UTP) [32].

Guanosine monophosphate reductase (GMPR) is a key enzyme in purine metabolism and catalyzes the NADPH-dependent conversion of GMP to IMP, thereby lowering intracellular guanine nucleotide concentrations and facilitating UCP1 activation [33,34,35]. Cold exposure drastically enhances GMPR expression in several organisms [34,36], and the functional interaction between GMPR-mediated degradation of brown adipocyte purine nucleotides and UCP1 activation has been clearly demonstrated in rats and mice [37,38].

There are two isoforms, GMPR1 and GMPR2, in mammals, exhibiting distinct expression patterns and regulatory roles. GMPR1 is enriched in the brain, testis, and prostate and has been linked to altered purine metabolism in Alzheimer’s disease [39], whereas GMPR2 is more broadly expressed in tissues such as the thyroid, kidney, liver, heart, and skeletal muscle, consistent with a housekeeping role in purine salvage and an additional function in cellular differentiation [35,40]. The presence of multiple GMPR isoforms—like multiple UCP isoforms—suggests functional specialization, enabling precise regulation of nucleotide balance, redox metabolism, and energy homeostasis across tissues and under varying physiological or environmental conditions in mammals. More broadly, GMPR activity has been implicated in stress responses, immune activation, and metabolic remodeling under conditions of energetic demand and disease [39]. Although UCP1 is absent in birds and reptiles, alternative thermogenic mechanisms involving purine metabolism have been proposed [41,42]. Comparative studies among cold-adapted vertebrates, including humans [43,44,45], rodents [34], cattle [46,47], amphibians [48] and arctic species [49,50], demonstrate that variations in GMPR and SLC25 expression contribute to physiological tolerance by enhancing mitochondrial efficiency, conserving energy, and coordinating metabolic and immune responses under environmental or physiological stress.

Emerging evidence suggests that corals may also retain homologs of these thermogenic and metabolic components. Similar to mammals, transcriptomic analyses have identified coral homologs of TRP channels involved in thermosensation [51,52,53], as well as genes related to neurotransmission, PKA signaling, lipolysis, and adenylate cyclase activity [54,55,56,57,58,59,60,61,62]. SLC25 genes, such as UCP2, UCP3, and UCP4, have been detected in the stony coral species *Porites lutea* [20], and UCP2/SLC25A8 in *Montastraea cavernosa* [61]. Because GMPR links nucleotide interconversion, NADPH utilization, and redox homeostasis, examining SLC25-related pathways provides an opportunity to explore whether corals possess analogous mechanisms for metabolic and redox balance during cold exposure. Here, we report the first expression, purification and characterization of recombinant GMPR from a coral species. We quantified the biochemical properties of PD GMPR, including kinetic parameters (Km, Vmax, kcat, and kcat/Km), and compared its activity with GMPRs from other organisms. Investigating GMPR function may assist in the long term to better understand cold-induced metabolic adaptation, potentially involving coordinating nucleotide balance and mitochondrial–cytosolic coupling. Such insights could inform strategies to enhance reef resilience under climate stress.

## 2. Materials and Methods

### 2.1. Materials

Sodium chloride (NaCl), Tris(hydroxymethyl)aminomethane (Tris), hydrochloric acid (HCl), sodium hydroxide (NaOH), disodium hydrogen phosphate (Na_2_HPO_4_), monosodium dihydrogen phosphate (NaH_2_PO_4_), ethylenediaminetetraacetic acid (EDTA), phenylmethylsulfonyl fluoride (PMSF), β-mercaptoethanol, Triton X-100, acetic acid (CH_3_COOH), ethanol, methanol, bromophenol blue, Coomassie Brilliant Blue G-250, potassium chloride (KCl), disodium hydrogen phosphate dihydrate (Na_2_HPO_4_·2H_2_O), dithiothreitol (DTT), fetal bovine serum (FBS), 100-fold concentrated stock solution of penicillin–streptomycin (Pen-Strep), bovine serum albumin (BSA), ethylenediaminetetraacetic acid (0.5 M EDTA), and phosphate-buffered saline (PBS) were purchased from Fisher Scientific, Pittsburgh, PA, USA. Monobasic potassium phosphate (KH_2_PO_4_) (G-biosciences), autoinduction medium, Terrific Broth (Boca Scientific, Dedham, MA, USA, Cat. No. GCM19.0500), protein molecular weight marker (Bio-Rad, Hercules, CA, USA, Cat. No. 1610374), HRP-conjugated mouse anti-His-tag monoclonal antibody (ABclonal, Woburn, MA, USA, Cat. No. AE028), Ni-NTA resin (Qiagen GmbH, Hilden, Nordrhein-Westfalen, Germany, Cat. No. 30210), protein assay kit (Pierce, Cat. No. 23225), 4–20% Mini PROTEAN TGX precast protein gel (Bio-Rad, Hercules, CA, USA, Cat. No. 4561094), PVDF membrane (Millipore-Sigma, Burlington, MA, USA, Cat. No. ISEQ85R), D-sorbitol (Sigma, Burlington, MA, USA, Cat. No. 240850) and imidazole (TCI, Portland, OR, USA, Cat. No. I0001) were purchased. Chemiluminescent reagent (Pierce, Dallas, TX, USA, Cat. No. 34580), guanosine monophosphate (GMP) and sucrose were purchased from (MP Biomedicals, Irvine, CA, USA, Cat # 101908 & 194018). NADPH and xanthosine monophosphate (XMP) (Combi-Blocks, San Diego, CA, USA, Cat. No. QV-9163) were used as indicated.

### 2.2. Expression

The gene encoding PD GMPR (genome ID: ASM370409v1; UniProt ID: A0A3M6UCF5) was cloned into the pET-28b(+) vector between NotI and NheI restriction sites by GenScript, Poscataway, NJ, USA. The recombinant plasmid was transformed into *E. coli* BL21 (DE3) competent cells (Novagen, Madison, WI, USA, Cat. No. 70956-3) via heat shock and plated on Luria–Bertani agar containing 50 μg/mL of kanamycin. This strain carries the λDE3 lysogen encoding T7 RNA polymerase under the lacUV5 promoter, with genotype F–ompT hsdSB (rB–mB–) gal dcm λ(DE3), enabling high-level expression from T7 promoter-driven vectors. A single colony was inoculated into 5 mL of autoinduction medium and grown overnight. This culture was used to inoculate 800 mL of autoinduction medium and incubated at 37 °C for 24 h at 200 rpm (final OD_600_ ≈ 1.2). Cells were harvested by centrifugation at 4000× *g* for 30 min. The resulting pellets were either used immediately or stored at −80 °C for subsequent protein extraction and purification.

### 2.3. Protein Extraction and Purification

#### 2.3.1. Protein Extraction of GMPR

A total of 7.7 g of bacterial pellet was resuspended in 110 mL of a 50 mM sodium phosphate buffer (pH 8.0), containing 300 mM NaCl, 1 mM PMSF, and 1% Triton X-100 (Fisher, Pittsburgh, PA, USA, Cat. No. C34H62011). The suspension was subjected to three freeze–thaw cycles, followed by centrifugation at 4000× *g* for 30 min.

#### 2.3.2. Purification

The clarified supernatant was applied to 1 mL of Ni-NTA resin to allow binding of His-tagged GMPR. The resin was washed with 30 column volumes of buffer (50 mM sodium phosphate (pH 8.0); 300 mM NaCl; and 0.05% Triton X-100). The bound protein was eluted using 250 mM imidazole (E1), followed by 300 mM imidazole (E2–E3). Elution fractions E2 and E3 were pooled, concentrated, and buffer-exchanged into 50 mM Tris (pH 8.0) with 0.05% Triton X-100. Protein purity was assessed using gel electrophoresis (SDS-PAGE) followed by Coomassie staining.

### 2.4. Biophysical Characterization

The UV absorption spectra of recombinant PD GMPR were recorded using a PerkinElmer (Shelton, CT, USA) Lambda 25 UV/VIS spectrophotometer at wavelengths ranging from 215 to 395 nm. Each reaction (500 μL) contained 50 mM Tris (pH 8.0), 1 mM EDTA, 1 μL of 5.3 μM purified GMPR, and 50 μM each of GMP or XMP and NADPH. Enzyme-only controls were pre-incubated at 37 °C for 10 min to establish equilibrium prior to substrate addition.

### 2.5. Circular Dichroism Spectroscopy

Circular dichroism (CD) measurements were performed on a Jasco J-815 CD spectrometer (Jasco Inc., Eaton, MD, USA) using PD GMPR at a final concentration of 0.5 mg/mL in 20 mM sodium phosphate buffer (pH 8.0), supplemented with 50 mM sodium chloride. Spectra were recorded in a 0.1 cm pathlength quartz cuvette over the wavelength range of 200–260 nm. Buffer baselines were collected under identical conditions and subtracted from all protein spectra prior to analysis. The experimental CD spectra for PD GMPR, along with the spectra calculated from the human GMPR1 and GMPR2 PDB structures, were analyzed using the BeStSel (Budapest, Hungary) secondary-structure deconvolution algorithm.

### 2.6. Enzyme Kinetics

#### 2.6.1. Kinetics Assay

Protein concentration was determined using the Pierce (Dallas, TX, USA) BCA Protein Assay Kit (Cat. No. 23225). GMPR activity was measured based on the reduction of GMP and the concomitant oxidation of NADPH by monitoring the decrease in absorbance at 340 nm over 30 min, using a BioTek Synergy H1 multimode reader, Winooski, VT, USA. Each reaction (200 μL) contained 50 mM Tris (pH 8.0), 1 mM EDTA, 1 μL of 5.3 μM purified GMPR, and varying concentrations of GMP or NADPH (50, 100, 250, or 500 μM) with fixed concentrations of NADPH (50, 100, or 150 μM) or GMP (25, 50, 75, or 100 μM). Enzyme-only controls were pre-incubated for 10 min at 37 °C to establish equilibrium before initiating reactions with the substrate.

#### 2.6.2. Kinetic Analysis

Enzymatic velocity was calculated by converting absorbance changes over time into concentration changes using Lambert–Beer’s law. Lineweaver–Burk plots (1/V vs. 1/[S]) were generated using Microsoft Excel. Linear regression was used to determine the best-fit line with R^2^ values > 0.9. Vmax was derived from the reciprocal of the y-intercept, and Km was calculated by multiplying the slope by Vmax for each substrate.

### 2.7. SDS-PAGE and Western Blot

#### 2.7.1. SDS-PAGE

Samples of 10 μL with varying concentration of protein (supernatant (1 μg), flow-through (1 μg), washes, and elution fractions (250 ng)) were mixed with 6X Laemmli buffer in a 1:6 (protein:buffer) ratio, boiled at 90 °C for 10 min, centrifuged at 4000× *g* for 10 min, and loaded onto SDS-PAGE (12% resolving; 4% stacking gel) using a Mini-Protean Cell System (Bio-Rad). Protein size was assessed using Bio-Rad’s pre-stained marker (Cat. No. 1610374). Electrophoresis was conducted at 120 V for 1 h. After separation, the proteins were visualized on one gel by Coomassie staining, while a duplicate gel was used for Western blotting. Whole-cell lysates were prepared in the same manner as described above to denature proteins and centrifuged briefly, and the supernatant was collected. Whole-cell lysate protein samples were separated on 4–20% Mini PROTEAN TGX precast protein gels, gradient SDS–PAGE gels in standard running buffer at 120 V for 90 min.

#### 2.7.2. Western Blotting

The protein bands were transferred onto a polyvinylidene difluoride membrane using a semi-dry transfer system (Bio-Rad Trans-Blot SD) at 20 V for 40 min. The membranes were blocked in 5% non-fat milk for 1 h at room temperature or overnight at 4 °C. HRP-conjugated mouse anti-His-tag monoclonal antibody (1:10,000 dilution in 5% milk) was incubated with the membrane for 2 h at room temperature or overnight at 4 °C. After three washes with a washing buffer (Tris-buffered saline with Tween-20) (10 min each), chemiluminescent substrate was added, and signals were detected using a Bio-Rad ChemiDoc MP Imaging System (Image Lab 5.0, Hercules, CA, USA).

The purified PD GMPR protein was transferred to the membrane by the gel–membrane sandwich immersion method and incubated with human polyclonal primary anti-GMPR1 antibody (rabbit, 1:500–1000; Sigma, Burlington, MA, USA, Cat. No. ABC945).

### 2.8. Bioinformatics

#### 2.8.1. Three-Dimensional Modeling and Visualization

The three-dimensional structure of PD GMPR was retrieved from AlphaFold2 and the AlphaFold Protein Structure Database interface [63]. The top-ranked model was selected based on predicted Local Distance Difference Test (pLDDT) scores. Crystal structures were available only for human GMPR1 and GMPR2 with pdb ids 2BLE and 2A7R [64], respectively. Protein structures were visualized using Pymol (version 2.5, Schrödinger LLC, New York, NY, USA) [65]. Active-site residues were labeled and color-coded consistently with those in the sequence alignment and 3D structural model, where each color represented a specific functional category of residues.

Similarly, the three-dimensional structure of pdam_00001745 was retrieved from AlphaFold DB [63]. Protein structures were visualized using Pymol (version 2.5, Schrödinger LLC, New York, NY, USA) [65].

#### 2.8.2. Alignment and Sequence Homology

Protein sequences were obtained from the uniprot database [64] for PD GMPR (A0A3M6UCF5), the 2 isoforms of GMPR in humans (P36959 and Q9P2T1), rats (Q9Z244 and A6KH36), bovine (Q08DA2 and Q32L93) and arctic fox from the NCBI database [66] (XP_041628038.1 and XP_041614815.1), and Western clawed frog (A9JRK1 and Q5BL91). Using the web-based NIH Constraint-Based Multiple Alignment Tool (COBALT) [67] alignment tool, the PD GMPR was aligned with the rest of the sequences. Once aligned, the COBALT provided homologous scores, which we used to calculate the percent similarity between the protein sequences. The isoelectric point (pI) and molecular weight (MW) of the protein sequence were determined using the ExPASy ProtParam web-based tool from Swiss Institute of Bioinformatics [68].

Similarly, the protein sequence of human UCP1 (UniProtKB: P25874) was used as a query in BLASTp (version 2.17.0, NCBI BLAST+ suite) searches against the PD proteome using NCBI BLAST+ suite, version 2.17.0 [66]. Multiple sequence alignments were performed using COBALT, implemented by NCBI [67], which incorporates pairwise sequence similarity and conserved domain constraints to improve alignment accuracy.

Long PD sequence identifiers related to UCPs were abbreviated by deleting the “pdam_0000” prefix and adding UCP, e.g., pdam_00001745 was referred to as UCP-1745.

#### 2.8.3. Phylogenetic and Distance Tree

Clustal Omega (version 1.0.2, EMBL-EBI, Hinxton, Cambridgeshire, UK) [69] was used to realign the same sequences obtained for the NIH COBALT alignment, given the need for the phylogenetic analysis. Based on the sequence similarities, Clustal Omega generated a phylogenetic tree for the group of aligned sequences using default parameters. Distance trees were generated using BLAST-based pairwise alignments, in which each database sequence was aligned independently to the human UCP1 query. Pairwise distances were calculated from query-based alignments using the Grishin model [70], and a neighbor-joining tree was constructed to visualize similarity relationships among mitochondrial carrier proteins. Sequences with insufficient overlap or excessive divergence (maximum sequence difference >0.85) were excluded [69,70].

#### 2.8.4. Root-Mean-Square Deviation (RMSD)

Structural alignments were carried out in Pymol (version 2.5, Schrödinger, LLC, New York, NY, USA). Each alignment of a protein sequence was compared with the PD predicted structure, and the root-mean-square deviation (RMSD) value, which corresponded to the average distances between equivalent atoms in the superimposed structures, was calculated.

## 3. Results

### 3.1. PD GMPR Expression and Purification

**Figure 1:**
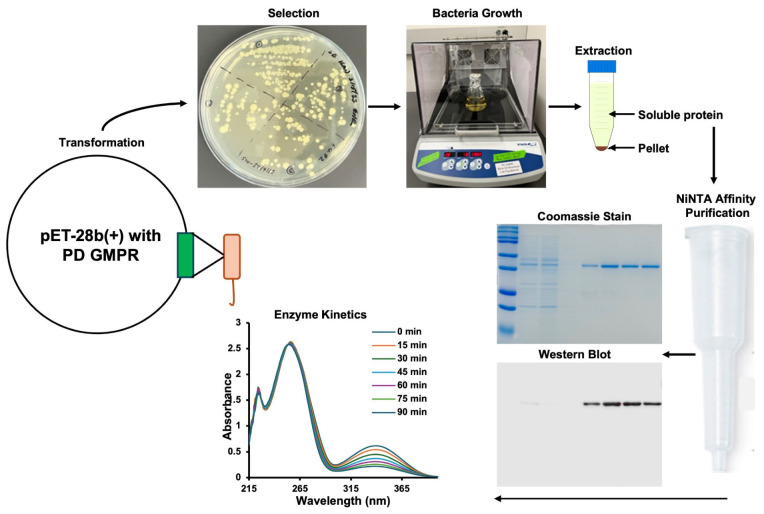
Overview of PD GMPR expression, purification, and characterization. The PD homologue of human GMPR was identified with Hhblits (version 3.3.0; Max Planck Institute for Developmental Biology: Tübingen, Germany), and the sequence was cloned into pET-28b(+) after codon optimization. The plasmid was transformed into *Escherichia coli* BL21 (DE3) and selected under kanamycin selection. Bacterial colonies were expanded into cultures and grown in autoinduction media. The protein was extracted from cells and purified using NiNTA affinity chromatography. Purity was evaluated with Coomassie staining, and identity was confirmed with anti-histidine Western blot. The purified protein was characterized by absorbance spectroscopy, which was also used to conduct enzyme kinetics analyses.

The recombinant PD GMPR gene, containing His-tags on both the C- and N-terminal, was cloned into the pET-28(b) vector and successfully transformed into *E. coli* BL21 (DE3) cells via heat shock. Protein expression and purification were achieved using Ni-NTA affinity chromatography, as detailed in Section 2.3 of Section 2. An overview of the expression, purification, and characterization workflow is presented in Figure 1.

The stepwise purification process of histidine-tagged PD GMPR using Ni-NTA resin is detailed in Section 2. Table 1 summarizes the total protein yield, specific activity, purification fold, and recovery yield. A 3.6-fold purification with a 30% final yield was achieved.

**Figure 2:**
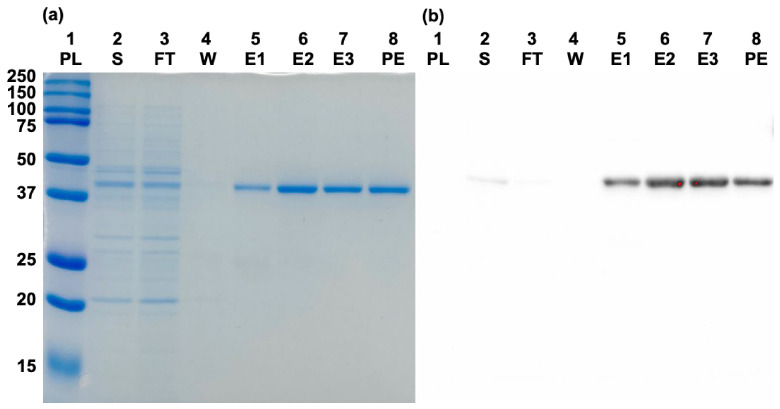
SDS-PAGE and Western blot analysis of PD GMPR. (**a**) Coomassie-stained gel; (**b**) Western blot. Lane 1: protein ladder (Bio-Rad #161037), lane 2: post-extraction supernatant, lane 3: flow-through, lane 4: wash, lanes 5–7: elution fractions E1–E3, and lane 8: pooled elution (E3–E4). For raw Western blot images and densitometry data for recombinant GMPR purification and validation please refer to Supplementary Figure S1.

The purity of the recombinant PD GMPR was assessed by SDS-PAGE and Coomassie Brilliant Blue staining. The samples used for the stained gel and Western blot were identical. The samples present were the supernatant, flow-through, wash, elution 1, elution 2, elution 3, and pooled elution in lanes 2–8, respectively. Lane 1 contained Bio-Rad’s pre-stained marker (Cat. No. 1610374) for molecular weight confirmation. The Coomassie-stained gel showed little to no contaminants in the purification elution lanes 5–8 (Figure 2a), indicative of a high purification percentage. The presence of the His-tag was confirmed via Western blotting using HRP-conjugated mouse anti-His-tag monoclonal antibody. The presence of purified GMPR was easily visualized and tagged with the antibody, shown in lanes 5–8 of the Western blot (Figure 2b). Recombinant PD GMPR showed a distinct band at approximately 38.97 kDa on both Coomassie-stained gels and His-tag Western blots, consistent with the predicted molecular weight and comparable to GMPR homologs reported in mammals. The final purified protein had a purity greater than 95%. The purified PD GMPR protein transferred to a PVDF membrane and probed with a human polyclonal anti-GMPR1 antibody in an independent Western blot analysis did not produce a detectable signal. However, the same sample showed clear bands when probed with an anti-His tag antibody.

### 3.2. Structural Prediction and Sequence Conservation Analysis

Human GMPR1 and GMPR2 are paralogous enzymes that share high sequence identity and a conserved (β/α)_8_ TIM-barrel fold, yet differ in structural dynamics, regulation, and physiological roles [42]. The first structure of human GMPR1 (PDB: 2BLE) revealed a tetrameric enzyme bound to GMP, clarifying substrate positioning, a catalytic cysteine nucleophile, and the mechanism of reductive deamination [71]. The first GMPR2 structure (PDB: 2A7R) demonstrated a similar tetrameric fold but with distinct loop conformations and active-site architecture, suggesting isoform-specific regulation [44]. GMPR2 in complex with IMP and NADPH in multiple conformations showed that cofactor mobility between “in” and “out” states is central to its catalytic cycle [72].

**Figure 3:**
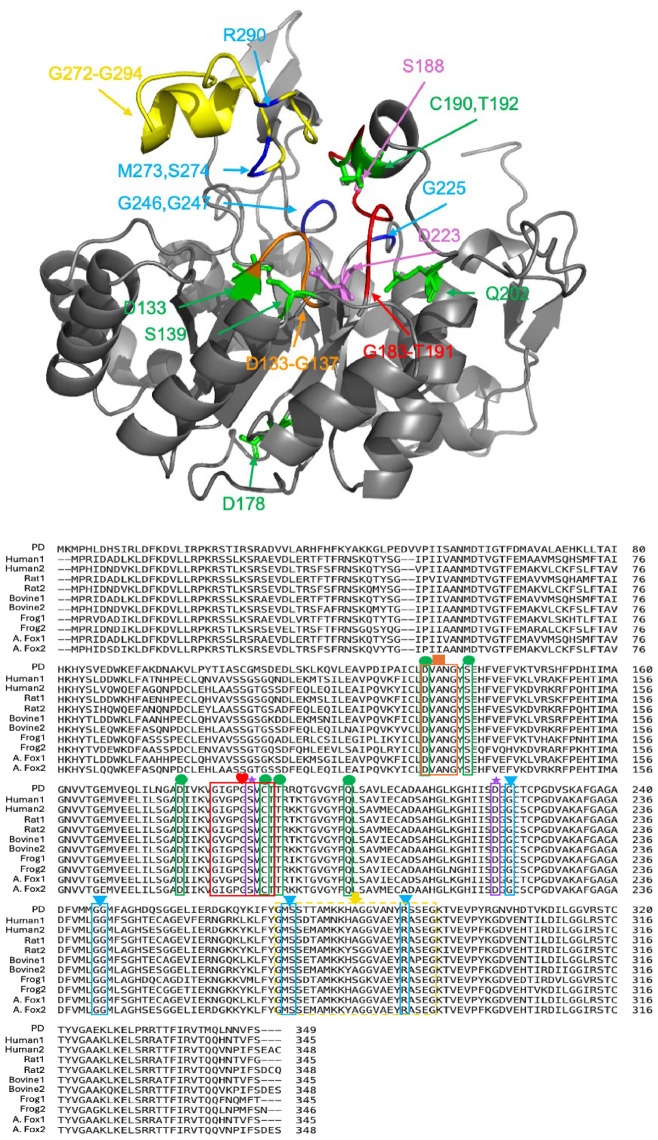
(**Top**) AlphaFold-predicted structure of PD GMPR. (**Bottom**) Multiple protein sequence alignment of PD GMPR with GMPR1 and GMPR2 from human, rat, bovine, Western clawed frog, and arctic fox. The colored residues in the structure model correspond to the colored residue boxes in the sequence alignment.

The AlphaFold-predicted tertiary structure of PD GMPR was visualized and edited using PyMOL (Figure 3, top). Given the NADPH-dependent reductive deamination mechanism converting GMP to IMP in mammalian GMPR, the functional residues and binding pocket for both GMP and NADPH are vital components for enzymatic activity. Using information from Martinelli et al. [33], the conserved functional residues most likely involved in GMPR’s enzymatic activity were highlighted in different colors. The conserved functional residues are color-coded to correspond with the sequence alignment in Figure 3 (bottom) and are located around the predicted GMP-binding pocket.

For the sequence alignment, species were selected based on phylogenetic relevance, ecological diversity, and availability of high-quality genomic data, ensuring a meaningful evolutionary and functional analysis (see Section 2). Multiple sequence alignment was performed between PD GMPR and GMPR1/2 isoforms from these species using the NIH COBALT web-based tool (Figure 3, bottom). The putative NADPH-binding site is boxed in orange with an orange square. The proposed active site loop is boxed in red with a red heart. A flexible loop hypothesized to act as a lid over bound GMP is outlined with a dashed yellow box and marked with a yellow arrow. Conserved catalytic residues are boxed in blue and marked with a blue dot; residues likely involved in substrate binding are boxed in green and marked with a green triangle; residues implicated in both catalysis and substrate binding are boxed in magenta with a star.

From CLUSTALW alignments, percent similarities were calculated for both interspecies and isoform comparisons (Table 2). Molecular weight and isoelectric point values were computed using the Expasy web-based tool. PD GMPR showed 70% sequence similarity with GMPR2 from human, rat, and arctic fox, and 68% similarity with the remaining sequences analyzed.

Structural comparisons were made using PyMOL with the crystal structures of human GMPR obtained from UniProt and AlphaFold-predicted structures for other species. The RMSD values are summarized in Table 3. PD GMPR showed the highest structural similarity to rat, bovine, and frog GMPR1/2 isoforms, with an RMSD of 0.2.

**Figure 4:**
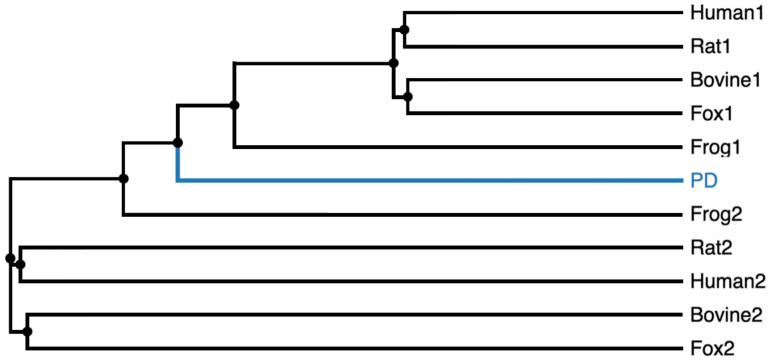
Phylogenetic tree of PD GMPR protein sequences. PD is highlighted in blue.

Phylogenetic analysis of aligned GMPR protein sequences was conducted using Clustal Omega (Figure 4). PD GMPR is highlighted in blue and clusters between GMPR1 and GMPR2 clades, supporting its evolutionary divergence and conserved functional role.

### 3.3. Spectral and Biophysical Characterization

**Figure 5:**
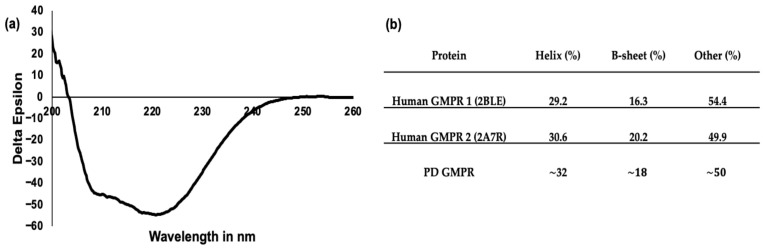
(**a**) Circular dichroism spectra of *PD* GMPR analyzed using the BeStSel algorithm. (**b**) The secondary-structure composition table which indicates a predominantly α/β architecture, with ~32% α-helix, ~18% β-sheet, and ~50% others. These values are consistent with a well-folded GMPR enzyme. CD spectra are shown as Delta Epsilon (∆ε) in M^−1^cm^−1^, also referred to molar absorptivity derived from the ellipticity, θ measured in units of mdeg through normalization with concentration and pathlength.

Figure 5 shows the circular dichroism characterization of PD GMPR (Figure 5a) and the predicted secondary structure composition extracted from the crystal structures of human GMPR1 and −2. We can see that the estimated secondary structure of PD GMPR is ~32%, which is highly consistent with the values from human GMPRs at 29% and 31% for GMPR1 and −2, respectively.

**Figure 6:**
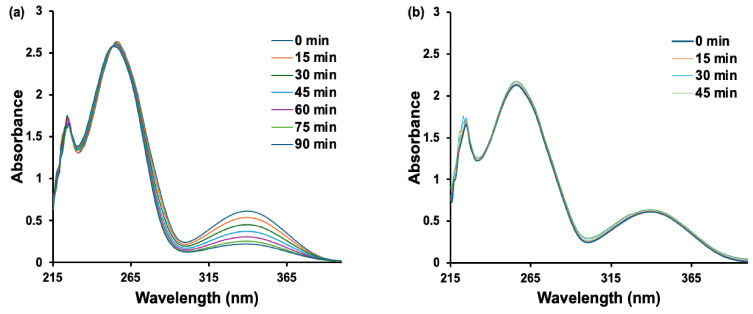
Absorption spectra of purified PD GMPR with GMP (**a**) and XMP (**b**).

UV absorption spectra of PD GMPR were recorded across 215–395 nm in the presence of GMP or XMP with NADPH. Each experiment was conducted for a minimum of 45 min to capture the change in absorbance at 340 nm. In the presence of GMP, there is a clear decrease in NADPH absorbance at 340 nm upon GMP binding, supporting GMP substrate specificity (Figure 6a). In the presence of XMP, there is no decrease in NADPH absorbance, indicative of competitive inhibition (Figure 6b).

### 3.4. Enzymatic Activity and Kinetics

**Figure 7:**
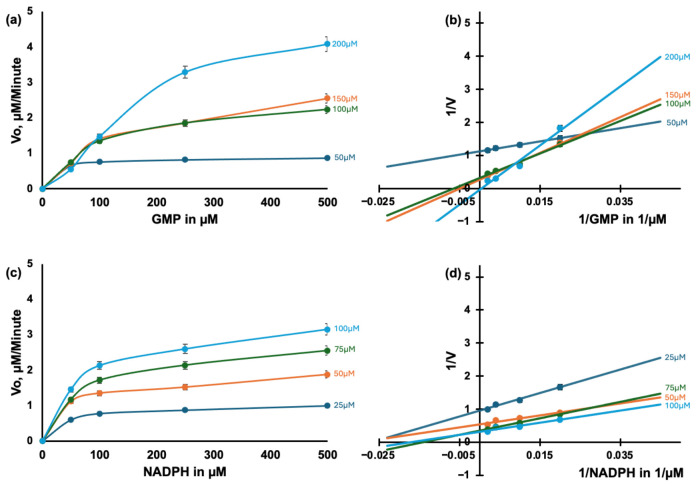
Kinetic analysis and Lineweaver–Burk plots for PD GMPR, generated using GraphPad Prism, version 11.0.0. (**a**) Initial velocity vs. GMP concentration at fixed NADPH. (**b**) Lineweaver–Burk plot (1/V vs. 1/[GMP]) at fixed NADPH. (**c**) Initial velocity vs. NADPH concentration at fixed GMP. (**d**) Lineweaver–Burk plot (1/V vs. 1/[NADPH]) at fixed GMP.

The kinetic analyses of human GMPRs indicate that both isoforms have comparable substrate affinities, though GMPR1 activity is more strongly modulated by allosteric effectors such as GTP (activator) and XMP (inhibitor), whereas GMPR2 relies more on conformational switching for turnover [73]. We quantified PD GMPR activity using a modified protocol adapted from Deng et al. [43]. Initial reaction velocities were measured under two experimental conditions: varying GMP concentrations at fixed NADPH levels (Figure 7a), and varying NADPH concentrations at fixed GMP levels (Figure 7c). Figure 7a shows the Michaelis–Menten enzyme rate plot for the reaction with fixed NADPH concentrations and varying GMP concentrations. The double-reciprocal Lineweaver–Burk plot of the fixed NADPH enzyme kinetics data is displayed in Figure 7b. Figure 7c shows the Michaelis–Menten enzyme rate plot for the reaction with fixed GMP concentrations and varying NADPH concentrations. The double-reciprocal Lineweaver–Burk plot of the fixed GMP enzyme kinetics data is displayed in Figure 7d.

In both experimental setups, the initial reaction velocity increased with the substrate concentration and approached a saturation plateau, consistent with Michaelis–Menten kinetics. When the GMP concentration was increased at constant NADPH levels (Figure 7a), the Michaelis–Menten curves exhibited elevated plateaus, indicating higher apparent Vmax values. This was corroborated by a downward shift in the y-intercept of the corresponding Lineweaver–Burk plot (Figure 7b), suggesting enhanced catalytic efficiency. Conversely, at lower GMP concentrations, such as 50 μM, the reaction curves were shallower, with a maximum plateau around 1 μM/min, and the Lineweaver–Burk plots showed higher y-intercepts over 1 min/μM compared with the other intercepts of <1 min/μM, indicative of reduced enzymatic turnover. A similar pattern emerged when NADPH concentrations were varied at fixed GMP levels (Figure 7c). Higher GMP concentrations led to increased Vmax values for NADPH, as evidenced by progressively elevated plateaus in the Michaelis–Menten curves. Across the full range of NADPH concentrations, reactions conducted at lower fixed GMP levels consistently exhibited diminished initial velocities, highlighting a substrate-limiting effect. This suggests that insufficient GMP availability imposes a kinetic bottleneck that cannot be compensated for by increasing NADPH concentration alone.

The Lineweaver–Burk plots for both substrate titrations (Figure 7b,d) revealed intersecting lines, indicative of a sequential (ordered) binding mechanism. This implies that the binding of one substrate may facilitate the binding and catalytic turnover of the other, consistent with cooperative substrate interaction. Such kinetic behavior aligns with previously reported trends for other NADPH-dependent enzymes, where substrate interplay modulates both affinity and catalytic efficiency [33,73].

The kinetic parameters of GMPR enzyme activity in PD were determined using Michaelis–Menten and Lineweaver–Burk analyses. The enzyme exhibited a Vmax of 1.85 ± 0.13 μM/min, a Km of 33.76 ± 6.44 μM, and a calculated kcat of 3.62 ± 0.25 /min for the fixed substrate GMP and a Vmax of 0.88 ± 0.01 μM/min, a Km of 17.71 ± 0.99 μM, and a kcat of 1.74 ± 0.02 /min for the fixed-substrate NADPH (Table 4). These values indicate moderate substrate affinity and relatively low catalytic efficiency. When compared with mammalian GMPRs, the PD enzyme displayed a ~5-fold lower kcat, suggesting slower turnover while maintaining comparable substrate affinity [44].

### 3.5. Phylogenetic Analysis of SLC25 Carrier Protein Candidates in PD

**Figure 8:**
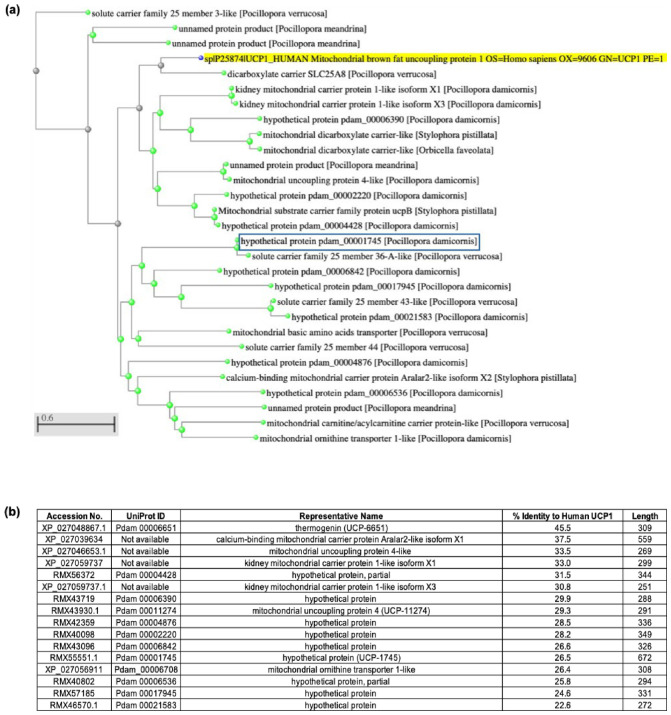
BLAST-based neighbor-joining distance tree of SLC25 proteins across stony corals constructed using human uncoupling protein 1 (UCP1; UniProtKB: P25874) as the query sequence. The highlighted UCP1 sequence (yellow) marks the query reference. (**a**) The tree resolves multiple SLC25 subfamilies, including UCP proteins and other mitochondrial carrier proteins. Branch nodes in green and branch lengths represent sequence similarity, and the scale bar in gray indicates substitutions per site. pdam_00001745 (blue box) is the largest PD protein. (**b**) Subset of 14 clusters of (**a**) containing PD sequences, with corresponding sequence lengths and percent sequence identity to human UCP1 (UniProtkB:P25874).

Since GMPR action tightly regulates uncoupling protein 1 (UCP1) activity in mammals, we investigated the possibility of such regulation in PD through homology detection (Figure 8). While UCP1 likely represents the evolution of specialized brown adipose tissue metabolism in mammals, other members of the broader solute carrier protein (SLC) mitochondrial carrier subfamily (SLC25) in *Pocillopora damicornis* (PD) may cooperate with GMPR in cold adaptation. Based on a recent phylogenetic analysis of UCP proteins in early-branching metazoans [74], it has been proposed that PD only contains two UCPs, one similar to UCP4, which we refer to as UCP-11274, and one unique to cnidarians (“cnUCP”), most closely related to UCP2/3, which we refer to as UCP-6651. The latter is the PD protein, with the highest sequence identity to UCP1 (Figure 8b). To investigate if there are potentially other related sequences in PD, we performed BLAST-based sequence similarity analysis using the human UCP1 protein as a query against PD and other stony coral proteomes. Distance tree analysis resolved 29 distinct sequence clusters, of which 14 clusters represent PD (Figure 8a). These clusters encompassed both UCP-like proteins and other metabolite carriers belonging to multiple SLC25 subfamilies. A table summarizing the predicted PD SLC25 family members is shown in Figure 8b, alongside the length of each sequence and the percent identity to human UCP1.

## 4. Discussion

This study presents the first biochemical characterization of GMPR in the reef-building coral, PD, a species known for its morphological plasticity and ecological resilience. By integrating biochemical, structural, and phylogenetic analyses, we provide compelling evidence that PD GMPR shares conserved features with mammalian GMPR isoforms while exhibiting distinct kinetic properties that may reflect coral-specific metabolic adaptations to cold stress. Corals are vulnerable to both heat and cold stress [12,13]. Recombinant PD GMPR was successfully expressed and purified, achieving >95% purity, a 3.6-fold purification enrichment, quantifying how many times purer GMPR was relative to the starting material; and 30% yield. Enzymatic assays confirmed NADPH-dependent reductive deamination of GMP to IMP, consistent with GMPR function in human, rat, and bacterial systems [34,43,44,75,76,77,78]. Absorption spectroscopy revealed a decrease in NADPH absorbance at 340 nm upon GMP binding, validating substrate specificity and confirming XMP as a competitive inhibitor [79,80,81]. Kinetic parameters indicate moderate substrate affinity and low catalytic efficiency, compared with mammalian GMPRs [82]. Relative to both human GMPR isoforms [42], PD GMPR displayed a modestly higher apparent Km for GMP (≈1.5–2-fold) and a substantially reduced catalytic turnover (≈4–5-fold), indicating that the coral enzyme requires higher substrate concentrations to reach half-maximal activity and operates with lower maximal flux. In contrast, PD GMPR exhibited increased sensitivity to NADPH, with an NADPH Km ~1.7–2-fold lower than that of human GMPR1 or GMPR2. This pattern suggests that PD GMPR activity is more tightly influenced by cellular reducing power than by GMP availability alone. Rather than implying an “energetically conservative” turnover rate, these kinetic differences may reflect distinct regulatory priorities in coral cells. A lower kcat combined with higher NADPH affinity is consistent with an enzyme whose flux is tuned to redox status, enabling purine interconversion to respond to shifts in NADPH/NADP^+^ balance [81,83]. Such a configuration could be advantageous under conditions where mitochondrial redox state fluctuates. Although the precise physiological role of PD GMPR remains to be established, the observed kinetic profile is compatible with a model in which GMPR contributes to maintaining nucleotide balance under variable redox conditions [42]. In this context, reduced catalytic flux may help prevent excessive GMP depletion, while heightened NADPH sensitivity could allow the enzyme to integrate purine metabolism with broader redox remodeling processes. These possibilities warrant further investigation but provide a mechanistically grounded interpretation of the kinetic distinctions between PD and human GMPRs. The reduced catalytic efficiency of PD GMPR likely reflects metabolic adjustments to its ecological niche as a symbiotic, reef-building coral inhabiting nutrient-limited environments. PD depends on algal photosynthates and tends to maintain slow, energetically conservative nucleotide turnover. Such adaptations may favor enzyme stability and regulation over rapid flux, consistent with coral energy conservation strategies under oligotrophic conditions. Structural divergence within active-site loops or cofactor-binding residues could further contribute to the lower turnover rate observed in PD GMPR. The phylogenetic analysis results positioned PD GMPR between mammalian GMPR1 and GMPR2 clades, suggesting that the coral enzyme may represent an ancestral or functionally intermediate form. This placement implies that GMPR diversification predates vertebrate lineage specialization and that PD GMPR retains conserved catalytic motifs with potential regulatory adaptations unique to marine invertebrates. In higher organisms, these functions became partitioned between GMPR1 and GMPR2, enabling localized, tissue-specific roles such as metabolic regulation. This intermediate placement suggests that GMPR isoform diversification and tissue-specific specialization may have occurred later during vertebrate evolution.

Analogous to the increase in GMPR expression found as a result of cold exposure in rats [34] and mice [37], we have proposed that a similar potential increase of GMPR in corals may also deplete intracellular guanine nucleotides, thereby potentially enhancing the activity of mitochondrial carriers with uncoupling or redox-regulatory potential. To look for candidates for such carriers in PD, we conducted phylogenetic analysis and identified several putative members of the SLC25 family in PD. The SLC25 family in human comprises 53 nuclear-encoded transporters and constitutes the largest family of inner mitochondrial membrane carriers, encompassing adenine nucleotide, aspartate–glutamate, phosphate, citrate, organic acid, and redox-related transporters [26,27]. Future work should aim to decipher what the specific roles of the putative PD SLC25 family members might be.

**Figure 9:**
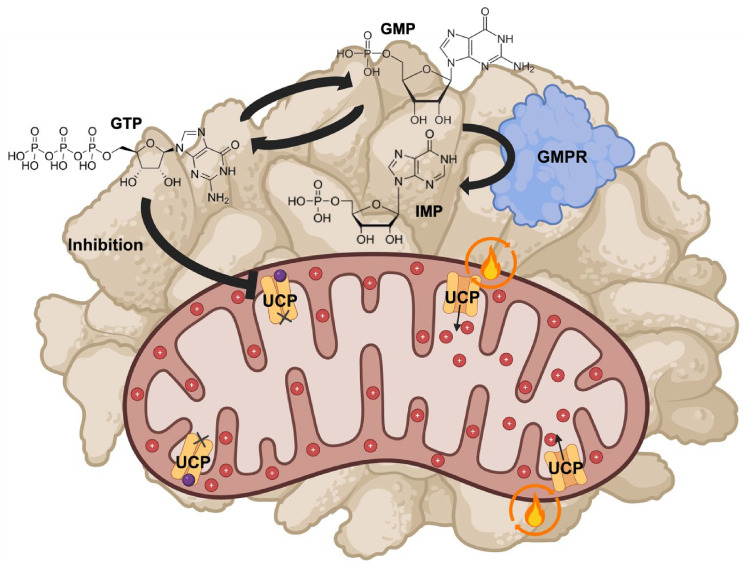
Proposed thermal regulation in PD mediated by GMPR activity. Schematic representation of the hypothesized metabolic mechanism linking guanosine monophosphate reductase (GMPR) activity to mitochondrial uncoupling during cold exposure in PD. GMPR catalyzes the NADPH-dependent conversion of GMP to IMP, thereby lowering intracellular GMP and GTP pools. Reduced GTP levels relieve nucleotide inhibition on uncoupling proteins (UCPs), facilitating proton leakage across the mitochondrial inner membrane and promoting localized heat dissipation or redox regulation. Cold stress and β-adrenergic-like signaling may enhance lipolysis, releasing free fatty acids that further activate UCPs via the cAMP–PKA pathway. Together, these processes may enable a thermogenic-like or redox-protective response in corals analogous to non-shivering thermogenesis in mammals. Created with BioRender.com.

Cold stress increases cellular energy demand, alters cytosolic NADH/NAD^+^ balance, and enhances amino acid catabolism and gluconeogenic flux [26,84,85,86,87]. How could GMPR and UCPs be involved in these processes? We, here, propose a model for thermoregulation in PD based on our findings and their analogy to mammals and other species (Figure 9). At the functional level, GMPR catalyzes the NADPH-dependent conversion of GMP to IMP, thereby regulating the balance between GMP and GTP. Purine nucleotides inhibit mitochondrial uncoupling proteins by binding to their nucleotide-regulatory sites [32]. In ectothermic organisms such as corals, similar purine-dependent regulation would more likely influence mitochondrial redox balance and metabolic efficiency rather than bulk heat production. Molecular simulations by Gagelin and Largeau et al. demonstrated that GDP and GTP occupy the same UCP1-binding pocket with differing phosphate interactions, where the triphosphate structure of GTP confers stronger inhibitory binding [86]. In mammals, cold exposure upregulates GMPR expression, lowering GMP and, consequently, GTP levels, which relieves GTP-based inhibition on UCP1 [34]. Because the identities of the specific PD UCPs or other SLC25 family members need to be more firmly established, we use “UCP” without subtype designation(s) in Figure 9. GMPR-mediated reduction in intracellular GTP levels could enhance UCP activity by alleviating nucleotide inhibition and, thus, activating carrier functions that allow adaptation to cold exposure, which may even involve mitochondrial uncoupling. Whether this occurs through a UCP-dependent or -independent mechanism remains to be determined, but both possibilities align with the notion of metabolic flexibility as a survival strategy under cold stress.

Gene expression studies support the existence of distinct molecular responses to cold and heat stress in corals. For instance, corals collected during summer and exposed to cold conditions (15 °C) showed upregulation of actin-related proteins and serine/threonine protein kinases, suggesting cytoskeletal remodeling and activation of signaling cascades associated with stress response [87]. In contrast, winter-collected corals exhibited minimal signs of cellular stress under the same cold exposure, indicating potential acclimatization to seasonal temperature variation. When subjected to heat stress, however, winter-collected corals upregulated phosphoenolpyruvate carboxykinase, a key enzyme in the gluconeogenic pathway, reflecting a metabolic shift to maintain energy balance under thermal challenge [87]. These contrasting gene expression patterns emphasize that coral response to cold and heat stress involves distinct molecular and metabolic adjustments aimed at preserving cellular integrity and redox equilibrium.

## 5. Conclusions

Cold stress during upwelling can induce rapid temperature fluctuations that may challenge coral–symbiont stability, analogous to how cold exposure in mammals activates thermogenic and redox-regulatory pathways to restore homeostasis. This study presents the first biochemical characterization of GMPR from PD, revealing conserved enzymatic properties and supporting a potential role in thermogenic-like responses to cold stress in corals. Structural, phylogenetic, and functional analyses suggest that PD GMPR retains features of an ancestral variant, providing a foundation from which GMPR1 and GMPR2 may have evolved to fulfill specialized regulatory and tissue-specific functions in complex organisms. Furthermore, we detected potential SLC family members, making it tempting to speculate that there may be ancestral metabolic mechanisms linking purine metabolism to mitochondrial functions, affording resilience through mitochondrial uncoupling and redox regulation. Metabolic resilience—the ability to maintain homeostasis and recover from environmental fluctuations—is central to coral adaptation. Responding to thermal stress requires coordinated metabolic, transcriptomic, and redox adjustments that sustain cellular function [88]. Such coordination, potentially mediated by GMPR and UCPs, may underlie resilience in PD during cold exposure. These findings provide insights into coral metabolic adaptation and may open avenues for enhancing coral reef resilience to environmental stressors through biochemical and molecular modulation of key metabolic pathways.

Future work should quantify GMPR and UCP homolog expression in PD under controlled cold exposure, investigate downstream signaling pathways linked to purine metabolism, mitochondrial regulation, and evaluate how GMPR activity influences mitochondrial respiration, reactive oxygen species production, and host–symbiont stability. In addition, cellular- and organismal-level studies assessing localization, thermal response, and metabolic flux will be essential to establish the functional relevance of GMPR and UCP proteins in coral physiology and thermotolerance. These multiscale approaches will help bridge the gap between in vitro enzyme characterization and in vivo thermotolerance.

## Figures and Tables

**Table 1:** Results of stepwise purification of histidine-tagged PD GMPR.

Purification Step	Total Protein (mg)	Total Enzyme Activity (U)	Specific Activity (U/mg)	Purification Fold	Yield in %
Crude extract	148	21,774	147	1	100
Pooled elution	12.3	6516	530	3.6	29.9

**Table 2:** GMPR protein homology across species. NCBI accession numbers for arctic fox and Uniprot IDs for all other species are provided. PDB identifiers of structures analyzed are given below the Uniprot ID for human GMPRs.

Species (ID)	PD A0A3M6UCF5	Human1 P36959 2BLE	Human2 Q9P2T1 2A7R	Rat1 Q9Z244	Rat2 A6KH36	Bovine1 Q08DA2	Bovine2 Q32L93	Frog1 A9JRK1	Frog2 Q5BL91	A.Fox1 XP_041628038.1	A.Fox2 XP_041614815.1
PD	100										
Human1	68	100									
Human2	70	80	100								
Rat1	68	96	80	100							
Rat2	70	79	96	79	100						
Bovine1	68	95	80	96	80	100					
Bovine2	68	79	95	79	94	79	100				
Frog1	68	83	80	83	80	83	80	100			
Frog2	68	79	85	78	84	79	84	80	100		
A.Fox1	68	95	81	95	79	97	80	83	80	100	
A.Fox2	70	80	97	80	95	81	97	81	86	81	100
Sequence length	349	345	348	345	348	340	348	345	346	345	348
Molecular weight (kDa)	38	37	38	37	38	37	38	37	38	37	38
Isoelectric point (pI)	7.7	6.6	6.8	6.5	7.1	6.2	7.1	7.6	7.1	6.9	6.6

**Table 3:** Root-mean-square deviation of GMPR structural alignments.

Species (ID)	PD A0A3M6UCF5	Human1 P36959	Human2 Q9P2T1	Rat1 Q9Z244	Rat2 A6KH36	Bovine1 Q08DA2	Bovine2 Q32L93	Frog1 A9JRK1	Frog2 Q5BL91
PD	0								
Human1	0.4	0							
Human2	0.5	0.4	0						
Rat1	0.2	0.4	0.5	0					
Rat2	0.2	0.4	0.4	0.1	0				
Bovine1	0.2	0.4	0.4	0.1	0.1	0			
Bovine2	0.2	0.4	0.4	0.1	0.1	0.1	0		
Frog1	0.2	0.4	0.4	0.1	0.1	0.1	0.1	0	
Frog2	0.2	0.4	0.4	0.2	0.1	0.1	0.1	0.1	0

**Table 4:** Kinetic parameters of GMPR enzyme activity in PD and humans. All available human data obtained from Deng et al. [42].

Species	Fixed Substrate	Conc. (μM)	Vmax (μM/min)	Km (μM)	kcat (1/min)	kcat/Km
PD	GMP	50	1.85 ± 0.13	33.76 ± 6.44	3.62 ± 0.25	0.11 ± 0.03
PD	NADPH	50	0.88 ± 0.01	17.71 ± 0.99	1.74 ± 0.02	0.09 ± 0.03
Human1	GMP	34	N/A	22.1 ± 2.2	17.04 ± 0.36	0.75
Human1	NADPH	70	N/A	34.8 ± 4.3	N/A	N/A
Human2	GMP	34	N/A	17.8 ± 3.5	15.90 ± 0.96	0.89
Human2	NADPH	70	N/A	29.3 ± 3.2	N/A	N/A

N/A corresponds to values that were unavailable at the time of this publication.

## Data Availability

The original contributions presented in this study are included in the article/Supplementary Materials. Further inquiries can be directed to the corresponding author.

## Supplementary Materials

The following supporting information can be downloaded at https://www.mdpi.com/article/10.3390/biology15110837/s1, Figure S1: Raw Western blot images and densitometry data for recombinant GMPR purification and validation.

## Abbreviations

The following abbreviations are used in this manuscript.

PD	*Pocillopora damicornis*
GMPR	Guanosine monophosphate reductase
GMP	Guanosine monophosphate
GDP	Guanosine diphosphate
GTP	Guanosine triphosphate
IMP	Inosine monophosphate
XMP	Xanthosine monophosphate
ATP	Adenosine triphosphate
CTP	Cytidine triphosphate
UTP	Uridine triphosphate
dTTP	Deoxythymidine triphosphate
UCP	Uncoupling protein
SLC25	Solute carrier family 25
AGC	Aspartate/glutamate carrier
NADPH	Nicotinamide adenine dinucleotide phosphate
RMSD	Root-mean-square deviation
TRP	Transient receptor potential protein
cAMP	Cyclic adenosine monophosphate
PKA	Protein kinase A
AMPK	AMP-activated protein kinase
Ni-NTA	Nickel–nitrilotriacetic acid
SDS-PAGE	Sodium dodecyl sulfate–polyacrylamide gel electrophoresis

## References

[1] Hoegh-Guldberg O., Mumby P.J., Hooten A.J., Steneck R.S., Greenfield P., Gomez E., Harvell C.D., Sale P.F., Edwards A.J., Caldeira K. (2007). Coral Reefs Under Rapid Climate Change and Ocean Acidification. Science.

[2] Hughes T.P., Kerry J.T., Álvarez-Noriega M., Álvarez-Romero J.G., Anderson K.D., Baird A.H., Babcock R.C., Beger M., Bellwood D.R., Berkelmans R. (2017). Global Warming and Recurrent Mass Bleaching of Corals. Nature.

[3] Intergovernmental Panel on Climate Change (IPCC) (2022). The Ocean and Cryosphere in a Changing Climate: Special Report of the Intergovernmental Panel on Climate Change.

[4] Putnam H.M., Gates R.D. (2015). Preconditioning in the Reef-Building Coral *Pocillopora damicornis* and the Potential for Trans-Generational Acclimatization in Coral Larvae under Future Climate Change Conditions. J. Exp. Biol..

[5] Schmidt-Roach S., Lundgren P., Miller K.J., Gerlach G., Noreen A.M.E., Andreakis N. (2013). Assessing Hidden Species Diversity in the Coral *Pocillopora damicornis* from Eastern Australia. Coral Reefs.

[6] Marhoefer S.R., Zenger K.R., Strugnell J.M., Logan M., Van Oppen M.J.H., Kenkel C.D., Bay L.K. (2021). Signatures of Adaptation and Acclimatization to Reef Flat and Slope Habitats in the Coral *Pocillopora damicornis*. Front. Mar. Sci..

[7] Veron J.E.N., Stafford-Smith M. (2000). Corals of the World.

[8] Edmunds P., Gates R. (2008). Acclimatization in Tropical Reef Corals. Mar. Ecol. Prog. Ser..

[9] García F.C., Osman E.O., Garcias-Bonet N., Delgadillo-Ordoñez N., Santoro E.P., Raimundo I., Villela H.D.M., Voolstra C.R., Peixoto R.S. (2024). Seasonal Changes in Coral Thermal Threshold Suggest Species-Specific Strategies for Coping with Temperature Variations. Commun. Biol..

[10] Lesser M.P. (2006). Oxidative Stress in Marine Environments: Biochemistry and Physiological Ecology. Annu. Rev. Physiol..

[11] Brown K.T., Genin A., Mello-Athayde M.A., Bergstrom E., Campili A., Chai A., Dove S.G., Ho M., Rowell D., Sampayo E.M. (2023). Marine Heatwaves Modulate the Genotypic and Physiological Responses of Reef-Building Corals to Subsequent Heat Stress. Ecol. Evol..

[12] Saxby T., Dennison W.C., Hoegh-Guldberg O. (2003). Photosynthetic Responses of the Coral *Montipora digitata* to Cold Temperature Stress. Mar. Ecol. Prog. Ser..

[13] Hoegh-Guldberg O., Fine M. (2004). Low Temperatures Cause Coral Bleaching. Coral Reefs.

[14] Roth M.S., Goericke R., Deheyn D.D. (2012). Cold Induces Acute Stress but Heat Is Ultimately More Deleterious for the Reef-Building Coral Acropora Yongei. Sci. Rep..

[15] de Barros Marangoni L.F., Rottier C., Ferrier-Pagès C. (2021). Symbiont Regulation in Stylophora Pistillata during Cold Stress: An Acclimation Mechanism against Oxidative Stress and Severe Bleaching. J. Exp. Biol..

[16] Rosales S.M., Huebner L.K., Evans J.S., Apprill A., Baker A.C., Becker C.C., Bellantuono A.J., Brandt M.E., Clark A.S., del Campo J. (2023). A Meta-Analysis of the Stony Coral Tissue Loss Disease Microbiome Finds Key Bacteria in Unaffected and Lesion Tissue in Diseased Colonies. ISME Commun..

[17] Jokiel P.L., Coles S.L. (1977). Effects of Temperature on the Mortality and Growth of Hawaiian Reef Corals. Mar. Biol..

[18] Bahr K.D., Jokiel P.L., Rodgers K.S. (2015). The 2014 Coral Bleaching and Freshwater Flood Events in Kāneʻohe Bay, Hawaiʻi. PeerJ.

[19] Takahashi S., Nakamura T., Sakamizu M., Woesik R.V., Yamasaki H. (2004). Repair Machinery of Symbiotic Photosynthesis as the Primary Target of Heat Stress for Reef-Building Corals. Plant Cell Physiol..

[20] Wei X., Yu K., Qin Z., Chen S., Pan N., Lan M. (2024). The Acute and Chronic Low-Temperature Stress Responses in Porites Lutea from a Relatively High-Latitude Coral Reef of the South China Sea. Front. Mar. Sci..

[21] Gong D., Lei J., He X., Hao J., Zhang F., Huang X., Gu W., Yang X., Yu J. (2024). Keys to the Switch of Fat Burning: Stimuli That Trigger the Uncoupling Protein 1 (UCP1) Activation in Adipose Tissue. Lipids Health Dis..

[22] Brand M.D., Esteves T.C. (2005). Physiological Functions of the Mitochondrial Uncoupling Proteins UCP2 and UCP3. Cell Metab..

[23] Sokolova I.M., Sokolov E.P. (2005). Evolution of Mitochondrial Uncoupling Proteins: Novel Invertebrate UCP Homologues Suggest Early Evolutionary Divergence of the UCP Family. FEBS Lett..

[24] Nikanorova A.A., Barashkov N.A., Pshennikova V.G., Nakhodkin S.S., Romanov G.P., Solovyev A.V., Fedorova S.A. (2025). The Evaluation of Significance of Uncoupling Protein Genes UCP1, UCP2, UCP3, UCP4, UCP5, and UCP6 in Human Adaptation to Cold Climates. Biology.

[25] Golozoubova V., Cannon B., Nedergaard J. (2006). UCP1 Is Essential for Adaptive Adrenergic Nonshivering Thermogenesis. Am. J. Physiol.-Endocrinol. Metab..

[26] Palmieri F. (2013). The Mitochondrial Transporter Family SLC25: Identification, Properties and Physiopathology. Mol. Asp. Med..

[27] Ruprecht J.J., Kunji E.R.S. (2020). The SLC25 Mitochondrial Carrier Family: Structure and Mechanism. Trends Biochem. Sci..

[28] Huang M., Yoo J.-K., Stickford A.S.L., Moore J.P., Hendrix J.M., Crandall C.G., Fu Q. (2021). Early Sympathetic Neural Responses during a Cold Pressor Test Linked to Pain Perception. Clin. Auton. Res..

[29] Braun K., Oeckl J., Westermeier J., Li Y., Klingenspor M. (2018). Non-Adrenergic Control of Lipolysis and Thermogenesis in Adipose Tissues. J. Exp. Biol..

[30] Louis S.N.S., Jackman G.P., Nero T.L., Iakovidis D., Louis W.J. (2000). Role of β-Adrenergic Receptor Subtypes in Lipolysis. Cardiovasc. Drugs Ther..

[31] Divakaruni A.S., Humphrey D.M., Brand M.D. (2012). Fatty Acids Change the Conformation of Uncoupling Protein 1 (UCP1). J. Biol. Chem..

[32] Jones S.A., Sowton A.P., Lacabanne D., King M.S., Palmer S.M., Zögg T., Pardon E., Steyaert J., Ruprecht J.J., Kunji E.R.S. (2025). Proton Conductance by Human Uncoupling Protein 1 Is Inhibited by Purine and Pyrimidine Nucleotides. EMBO J..

[33] Martinelli L.K.B., Ducati R.G., Rosado L.A., Breda A., Selbach B.P., Santos D.S., Basso L.A. (2011). Recombinant Escherichia Coli GMP Reductase: Kinetic, Catalytic and Chemical Mechanisms, and Thermodynamics of Enzyme–Ligand Binary Complex Formation. Mol. Biosyst..

[34] Salvatore D., Bartha T., Larsen P.R. (1998). The Guanosine Monophosphate Reductase Gene Is Conserved in Rats and Its Expression Increases Rapidly in Brown Adipose Tissue during Cold Exposure. J. Biol. Chem..

[35] Bast-Habersbrunner A., Fromme T. (2020). Purine Nucleotides in the Regulation of Brown Adipose Tissue Activity. Front. Endocrinol..

[36] Ansaloni F., Gerdol M., Torboli V., Fornaini N.R., Greco S., Giulianini P.G., Coscia M.R., Miccoli A., Santovito G., Buonocore F. (2021). Cold Adaptation in Antarctic Notothenioids: Comparative Transcriptomics Reveals Novel Insights in the Peculiar Role of Gills and Highlights Signatures of Cobalamin Deficiency. Int. J. Mol. Sci..

[37] Fromme T., Kleigrewe K., Dunkel A., Retzler A., Li Y., Maurer S., Fischer N., Diezko R., Kanzleiter T., Hirschberg V. (2018). Degradation of Brown Adipocyte Purine Nucleotides Regulates Uncoupling Protein 1 Activity. Mol. Metab..

[38] Keebaugh A.C., Thomas J.W. (2010). The Evolutionary Fate of the Genes Encoding the Purine Catabolic Enzymes in Hominoids, Birds, and Reptiles. Mol. Biol. Evol..

[39] Liu H., Luo K., Luo D. (2018). Guanosine Monophosphate Reductase 1 Is a Potential Therapeutic Target for Alzheimer’s Disease. Sci. Rep..

[40] Uhlén M., Fagerberg L., Hallström B.M., Lindskog C., Oksvold P., Mardinoglu A., Sivertsson Å., Kampf C., Sjöstedt E., Asplund A. (2015). Tissue-Based Map of the Human Proteome. Science.

[41] Tattersall G.J. (2016). Reptile Thermogenesis and the Origins of Endothermy. Zoology.

[42] Deng Y., Wang Z., Ying K., Gu S., Ji C., Huang Y., Gu X., Wang Y., Xu Y., Li Y. (2002). NADPH-Dependent GMP Reductase Isoenzyme of Human (GMPR2). Int. J. Biochem. Cell Biol..

[43] Li J., Wei Z., Zheng M., Gu X., Deng Y., Qiu R., Chen F., Ji C., Gong W., Xie Y. (2006). Crystal Structure of Human Guanosine Monophosphate Reductase 2 (GMPR2) in Complex with GMP. J. Mol. Biol..

[44] Spector T., Jones T.E., Miller R.L. (1979). Reaction Mechanism and Specificity of Human GMP Reductase. Substrates, Inhibitors, Activators, and Inactivators. J. Biol. Chem..

[45] Diao Z., Shimokawa F., Yoshioka H., Itoyama E., Matsumura M., Murakami M., Kitamura S., Nagase H., Matsui T., Funaba M. (2023). Possibility of Uncoupling Protein 1 Expression in Bovine Fast-Twitch Muscle Fibers. J. Vet. Med. Sci..

[46] Arfuso F., Arrigo F., Rizzo M., Sisia G., Fiore E., Giannetto C., Liotta L., Piccione G., Lopreiato V. (2025). Investigating Mitochondrial Uncoupling Protein 1 and Leptin in the Interplay of Metabolic Adaptation and Inflammatory Response of Dairy Cows during the Peripartum Period. Front. Vet. Sci..

[47] Kayes S.M., Cramp R.L., Hudson N.J., Franklin C.E. (2009). Surviving the Drought: Burrowing Frogs Save Energy by Increasing Mitochondrial Coupling. J. Exp. Biol..

[48] Lord E., Marangoni A., Baca M., Popović D., Goropashnaya A.V., Stewart J.R., Knul M.V., Noiret P., Germonpré M., Jimenez E.-L. (2022). Population Dynamics and Demographic History of Eurasian Collared Lemmings. BMC Ecol. Evol..

[49] Lynch V.J., Bedoya-Reina O.C., Ratan A., Sulak M., Drautz-Moses D.I., Perry G.H., Miller W., Schuster S.C. (2015). Elephantid Genomes Reveal the Molecular Bases of Woolly Mammoth Adaptations to the Arctic. Cell Rep..

[50] Burns D., Venditti V., Potoyan D.A. (2023). Temperature Sensitive Contact Modes Allosterically Gate TRPV3. PLoS Comput. Biol..

[51] Bernardet C., Tambutté E., Techer N., Tambutté S., Venn A.A. (2019). Ion Transporter Gene Expression Is Linked to the Thermal Sensitivity of Calcification in the Reef Coral *Stylophora pistillata*. Sci. Rep..

[52] Sun Y., Lan Y., Rädecker N., Sheng H., Diaz-Pulido G., Qian P.-Y., Huang H. (2024). Gene Expression of *Pocillopora damicornis* Coral Larvae in Response to Acidification and Ocean Warming. BMC Genom. Data.

[53] Kaniewska P., Chan C.-K.K., Kline D., Ling E.Y.S., Rosic N., Edwards D., Hoegh-Guldberg O., Dove S. (2015). Transcriptomic Changes in Coral Holobionts Provide Insights into Physiological Challenges of Future Climate and Ocean Change. PLoS ONE.

[54] Anctil M., Hayward D.C., Miller D.J., Ball E.E. (2007). Sequence and Expression of Four Coral G Protein-Coupled Receptors Distinct from All Classifiable Members of the Rhodopsin Family. Gene.

[55] Murthy M.H.S., Jasbi P., Lowe W., Kumar L., Olaosebikan M., Roger L., Yang J., Lewinski N., Daniels N., Cowen L. (2024). Insulin Signaling and Pharmacology in Humans and in Corals. PeerJ.

[56] Kenkel C.D., Sheridan C., Leal M.C., Bhagooli R., Castillo K.D., Kurata N., McGinty E., Goulet T.L., Matz M.V. (2014). Diagnostic Gene Expression Biomarkers of Coral Thermal Stress. Mol. Ecol. Resour..

[57] Imbs A.B., Yakovleva I.M. (2012). Dynamics of Lipid and Fatty Acid Composition of Shallow-Water Corals under Thermal Stress: An Experimental Approach. Coral Reefs.

[58] Imbs A.B., Dembitsky V.M. (2023). Coral Lipids. Mar. Drugs.

[59] Nielsen D.A., Petrou K. (2023). Lipid Stores Reveal the State of the Coral-Algae Symbiosis at the Single-Cell Level. ISME Commun..

[60] Rosic N., Ling E.Y.S., Chan C.-K.K., Lee H.C., Kaniewska P., Edwards D., Dove S., Hoegh-Guldberg O. (2015). Unfolding the Secrets of Coral–Algal Symbiosis. ISME J..

[61] Beavers K.M., Gutierrez-Andrade D., Van Buren E.W., Emery M.A., Brandt M.E., Apprill A., Mydlarz L.D. (2025). Machine Learning Reveals Distinct Gene Expression Signatures across Tissue States in Stony Coral Tissue Loss Disease. R. Soc. Open Sci..

[62] Słocińska M., Barylski J., Jarmuszkiewicz W. (2016). Uncoupling Proteins of Invertebrates: A Review. IUBMB Life.

[63] Jumper J., Evans R., Pritzel A., Green T., Figurnov M., Ronneberger O., Tunyasuvunakool K., Bates R., Žídek A., Potapenko A. (2021). Highly Accurate Protein Structure Prediction with AlphaFold. Nature.

[64] Bateman A., Martin M.-J., Orchard S., Magrane M., Adesina A., Ahmad S., Bowler-Barnett E.H., Bye-A-Jee H., Carpentier D., The UniProt Consortium (2025). UniProt: The Universal Protein Knowledgebase in 2025. Nucleic Acids Res..

[65] DeLano W.L. (2002). The PyMOL Molecular Graphics System.

[66] Sayers E.W., Beck J., Bolton E.E., Brister J.R., Chan J., Connor R., Feldgarden M., Fine A.M., Funk K., Hoffman J. (2025). Database Resources of the National Center for Biotechnology Information in 2025. Nucleic Acids Res..

[67] Papadopoulos J.S., Agarwala R. (2007). COBALT: Constraint-Based Alignment Tool for Multiple Protein Sequences. Bioinformatics.

[68] Duvaud S., Gabella C., Lisacek F., Stockinger H., Ioannidis V., Durinx C. (2021). Expasy, the Swiss Bioinformatics Resource Portal, as Designed by Its Users. Nucleic Acids Res..

[69] Sievers F., Wilm A., Dineen D., Gibson T.J., Karplus K., Li W., Lopez R., McWilliam H., Remmert M., Söding J. (2011). Fast, Scalable Generation of High-quality Protein Multiple Sequence Alignments Using Clustal Omega. Mol. Syst. Biol..

[70] Cai W., Pei J., Grishin N.V. (2004). Reconstruction of Ancestral Protein Sequences and Its Applications. BMC Evol. Biol..

[71] Bunkoczi G., Haroniti A., Ng S., Von Delft F., Oppermann U., Arrowsmith C., Sundstrom M., Edwards A., Gileadi O. (2005). Structure of Human Guanosine Monophosphate Reductase GMPR1 in Complex with GMP: 2ble.

[72] Patton G.C., Stenmark P., Gollapalli D.R., Sevastik R., Kursula P., Flodin S., Schuler H., Swales C.T., Eklund H., Himo F. (2011). Cofactor Mobility Determines Reaction Outcome in the IMPDH and GMPR (β-α)8 Barrel Enzymes. Nat. Chem. Biol..

[73] Dobie F., Berg A., Boitz J.M., Jardim A. (2007). Kinetic Characterization of Inosine Monophosphate Dehydrogenase of Leishmania Donovani. Mol. Biochem. Parasitol..

[74] Gamero-Mora E., Muhlia-Almazán A. (2025). The Mitochondrial Uncoupling Proteins in Early-Branching Animals: Comparative Analysis and Transcriptional Response to Temperature in the Jellyfish Stomolophus Sp.2. J. Bioenerg. Biomembr..

[75] Lee Y., Willers C., Kunji E.R.S., Crichton P.G. (2015). Uncoupling Protein 1 Binds One Nucleotide per Monomer and Is Stabilized by Tightly Bound Cardiolipin. Proc. Natl. Acad. Sci. USA.

[76] Andrews S.C., Guest J.R. (1988). Nucleotide Sequence of the Gene Encoding the GMP Reductase of *Escherichia coli*. Biochem. J..

[77] Stephens R.W., Whittaker V.K. (1973). Calf Thymus GMP Reductase: Control by XMP. Biochem. Biophys. Res. Commun..

[78] Doležal M., Knejzlík Z., Kouba T., Filimoněnko A., Šváchová H., Dedola M., Klíma M. (2026). Structural Basis for Allosteric Regulation of Mycobacterial Guanosine 5′-Monophosphate Reductase by ATP and GTP. Nat. Commun..

[79] Struntz N.B., Hu T., White B.R., Olson M.E., Harki D.A. (2012). Inhibition of Guanosine Monophosphate Synthetase by the Substrate Enantiomer L-XMP. ChemBioChem.

[80] Bessho T., Okada T., Kimura C., Shinohara T., Tomiyama A., Imamura A., Kuwamura M., Nishimura K., Fujimori K., Shuto S. (2016). Novel Characteristics of Trypanosoma Brucei Guanosine 5′-Monophosphate Reductase Distinct from Host Animals. PLoS Negl. Trop. Dis..

[81] Ju H.-Q., Lin J.-F., Tian T., Xie D., Xu R.-H. (2020). NADPH Homeostasis in Cancer: Functions, Mechanisms and Therapeutic Implications. Signal Transduct. Target. Ther..

[82] Tavoulari S., Lacabanne D., Pereira G.C., Thangaratnarajah C., King M.S., He J., Chowdhury S.R., Tilokani L., Palmer S.M., Prudent J. (2024). Distinct Roles for the Domains of the Mitochondrial Aspartate/Glutamate Carrier Citrin in Organellar Localization and Substrate Transport. Mol. Metab..

[83] Lane A.N., Fan T.W.-M. (2015). Regulation of Mammalian Nucleotide Metabolism and Biosynthesis. Nucleic Acids Res..

[84] del Arco A., Contreras L., Pardo B., Satrustegui J. (2016). Calcium Regulation of Mitochondrial Carriers. Biochim. Biophys. Acta BBA-Mol. Cell Res..

[85] Haman F., Péronnet F., Kenny G.P., Massicotte D., Lavoie C., Scott C., Weber J.-M. (2002). Effect of Cold Exposure on Fuel Utilization in Humans: Plasma Glucose, Muscle Glycogen, and Lipids. J. Appl. Physiol..

[86] Gagelin A., Largeau C., Masscheleyn S., Piel M.S., Calderón-Mora D., Bouillaud F., Hénin J., Miroux B. (2023). Molecular Determinants of Inhibition of UCP1-Mediated Respiratory Uncoupling. Nat. Commun..

[87] Lee S.T.M., Keshavmurthy S., Fontana S., Takuma M., Chou W.-H., Chen C.A. (2018). Transcriptomic Response in Acropora Muricata under Acute Temperature Stress Follows Preconditioned Seasonal Temperature Fluctuations. BMC Res. Notes.

[88] Jasbi P., Mohr A.E., Murthy M.H.S., Klein-Seetharaman J. (2025). Understanding Metabolic Resilience by Unraveling Temporal Dynamics of Cellular Responses. Trends Endocrinol. Metab..

