# Supplementary material for: Characterization of Recombinant GMPR from Pocillopora damicornis and Potential Mechanisms of Cold-Induced Metabolic Adaptation
Source: Biology (Basel). 2026 May 27;15(11):837. doi: 10.3390/biology15110837 (PMC13256074; doi:10.3390/biology15110837)
Supplement: Supplementary file 1 [file biology-15-00837-s001.zip › biology-4002592-supplementary.pdf]

Supplementary Figure S1. Raw Western blot images and densitometry data for recombinant GMPR purification andd validation.

(a) Coomassie-stained SDS–PAGE gel showing purified recombinant PD GMPR protein (MW=~38.97). Approximately 10 μL of protein was loaded per lane (E1–E3 = elution fractions; P = pooled elution), and molecular-weight standard was used as reference.
(b) Raw Western blot image confirming His-tagged PD GMPR detection; lanes correspond to elution fractions as shown in (a).
(c) Densitometric analysis of Western blot bands used to calculate relative intensity values for each elution fraction. Intensity was determined using ImageJ software, normalized to background, and reported as arbitrary units.

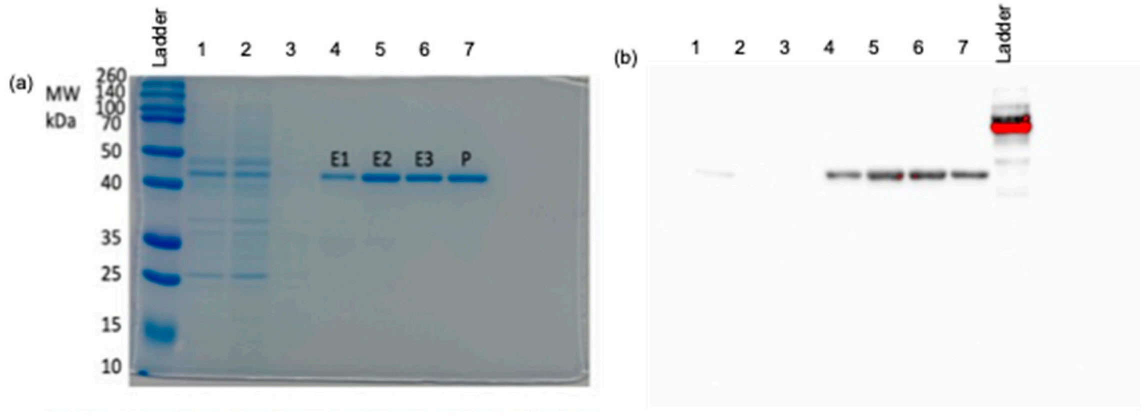

(c)

Lanes	GMPR Purification	Band intensity
Lane1	Supernatant	5.857
Lane2	Flow Through	1.061
Lane3	Wash	0.061
Lane4	Elution 1	51.724
Lane5	Elution 2	86.095
Lane6	Elution 3	87.006
Lane7	Pooled Elution	67.47
